# Redox mechanisms of environmental toxicants on male reproductive function

**DOI:** 10.3389/fcell.2024.1333845

**Published:** 2024-02-26

**Authors:** Tarique Hussain, Elsayed Metwally, Ghulam Murtaza, Dildar Hussain Kalhoro, Muhammad Ismail Chughtai, Bie Tan, Ali Dogan Omur, Shakeel Ahmed Tunio, Muhammad Shahzad Akbar, Muhammad Saleem Kalhoro

**Affiliations:** ^1^ College of Animal Science and Technology, Hunan Agricultural University, Changsha, Hunan, China; ^2^ Animal Science Division, Nuclear Institute for Agriculture and Biology College, Pakistan Institute of Engineering and Applied Sciences (NIAB-C, PIEAS), Faisalabad, Pakistan; ^3^ Department of Cytology and Histology, Faculty of Veterinary Medicine, Suez Canal University, Ismailia, Egypt; ^4^ Department of Livestock and Fisheries, Government of Sindh, Karachi, Pakistan; ^5^ Department of Veterinary Microbiology, Faculty of Animal Husbandry and Veterinary Sciences, Sindh Agriculture University, Tandojam, Sindh, Pakistan; ^6^ Department of Artificial Insemination, Faculty, Veterinary Medicine, Ataturk University, Erzurum, Türkiye; ^7^ Department of Livestock Management, Faculty of Animal Husbandry and Veterinary Sciences, Sindh Agriculture University, Tandojam, Sindh, Pakistan; ^8^ Faculty of Animal Husbandry and Veterinary Sciences, University of Poonch, Rawalakot, Pakistan; ^9^ Department of Agro-Industrial, Food, and Environmental Technology, Faculty of Applied Science, Food and Agro-Industrial Research Centre, King Mongkut’s University of Technology North Bangkok, Bangkok, Thailand

**Keywords:** environmental toxicants, male reproduction, sperm quality and reactive oxygen species, endocrine disruptors, redox mechanisms

## Abstract

Humans and wildlife, including domesticated animals, are exposed to a myriad of environmental contaminants that are derived from various human activities, including agricultural, household, cosmetic, pharmaceutical, and industrial products. Excessive exposure to pesticides, heavy metals, and phthalates consequently causes the overproduction of reactive oxygen species. The equilibrium between reactive oxygen species and the antioxidant system is preserved to maintain cellular redox homeostasis. Mitochondria play a key role in cellular function and cell survival. Mitochondria are vulnerable to damage that can be provoked by environmental exposures. Once the mitochondrial metabolism is damaged, it interferes with energy metabolism and eventually causes the overproduction of free radicals. Furthermore, it also perceives inflammation signals to generate an inflammatory response, which is involved in pathophysiological mechanisms. A depleted antioxidant system provokes oxidative stress that triggers inflammation and regulates epigenetic function and apoptotic events. Apart from that, these chemicals influence steroidogenesis, deteriorate sperm quality, and damage male reproductive organs. It is strongly believed that redox signaling molecules are the key regulators that mediate reproductive toxicity. This review article aims to spotlight the redox toxicology of environmental chemicals on male reproduction function and its fertility prognosis. Furthermore, we shed light on the influence of redox signaling and metabolism in modulating the response of environmental toxins to reproductive function. Additionally, we emphasize the supporting evidence from diverse cellular and animal studies.

## Introduction

Environmental chemicals, such as pesticides, are widely used in agriculture and public health protection programs (Hoffman et al., 2000). Over 11,000 pesticides are commercially marketed in European countries (Jeong et al., 2012; Darko et al., 2017) for different purposes. The worldwide usage of pesticides is mainly applied to maximize yield, although they also contaminate food, water, and the environment (El-Nahhal, 2004). Many pesticides have been reported to have detrimental effects on human health and cause environmental problems (Organization, 1990; Alewu and Nosiri, 2011). However, some of them are banned by regulating authorities (Alewu and Nosiri, 2011). The interaction of pesticides with humans and animals occurs via different routes, such as skin contact, ingestion, and inhalation. Some precautionary measures must be considered, such as the pesticide group, length and exposure route, and individual status. After ingestion, the pesticide can be metabolized, eliminated, and finally stored in body fat (Organization, 1990; Alewu and Nosiri, 2011). The negative impact of pesticides has been reported on health, causing reproductive, hormonal, and other problems (Sanborn et al., 2007; Mnif et al., 2011), while the residues of pesticides have been documented in various food items and animal feeds (Chourasiya et al., 2015). It is worth noting that various practices cannot remove pesticide residues (Reiler et al., 2015), and in many cases, the concentration does not exceed the safe limits (Nougadère et al., 2012; Reiler et al., 2015). However, a safe limit may also pose a threat to health due to the composition of more than one chemical substance with synergistic effects (Organization, 1990; Lu et al., 2015). Evidence of pesticide residues has also been documented in human breast milk, possibly due to prenatal exposure (Damgaard et al., 2006; Lu et al., 2015).

Evidence has shown that numerous pesticides and their metabolites can be considered entities that can disrupt endocrine functions (Zhang et al., 2016). Such interactions can influence normal physiology and negatively impact developmental, reproductive, endocrine, and other systems (Sathyanarayana et al., 2012; Büyükgebiz, 2015). Moreover, chlorinated compounds are known as endocrine-activating pesticides, for instance, hexachlorobenzene (HCB), hexachlorocyclohexane (HCH), dichlorodiphenyltrichloroethane (DDT), and its metabolite, dichlorodiphenyldichloroethylene (DDE). Current epidemiological and other aspects have documented higher evidence of male reproductive issues associated with cancer in the testicles, lower sperm production, and other malformations in the genitourinary system (Mendiola et al., 2014). The latter anomalies disrupt the endocrine system due to the use of pesticides in agriculture and, in turn, cause cryptorchidism (Weidner et al., 1998; Fernandez et al., 2007), hypospadias (Kristensen et al., 1997; Kraft et al., 2010), and micropenis (Gaspari et al., 2011; Gaspari et al., 2012). Collectively, these aberrations make organisms susceptible to testicular dysgenesis syndrome (Skakkebaek et al., 2001).

Phthalates are chemicals widely used as plasticizers in consumer products. Their enormous usage has drawn attention to their health hazard effects. The literature has demonstrated that phthalate exposure causes various disorders that significantly affect reproductive function (Benjamin et al., 2017). A serious health concern is that reproductive problems are increasing worldwide, such as cancers due to hormones. Back in 2015, approximately 12% of couples had infertility issues globally, which resulted in reduced fecundity (Kumar and Singh, 2015). During the last five decades, the sperm concentration has been reduced to 32.5%. The main cause of reproductive problems may be due to phthalates (Sengupta et al., 2018). There is a dire need to focus on fertility-related problems by better understanding of the phthalate mechanism and finding possible mitigation approaches.

Environmental toxicants augment cellular and molecular mechanisms that may vary with age, chronicity, and dose of exposure. Mitochondria are the central part responsible for regulating several functions of metabolic and cellular signaling, which coordinate to maintain cell survival and homeostasis. Disruption in the biological system due to environmental toxicants influences these events and eventually causes adverse effects (Duarte-Hospital et al., 2022). The impact of environmental toxicants on male reproduction is illustrated in Table 1.

**TABLE 1:** Effect of environmental toxicants on male reproduction.

Toxicant	Toxic effect	Animal model	Reference
2,2-Bis(4-chlorophenyl)-1,1-dichloroethylene (DDE)	Impairment of mitochondrial function in the testis	Rat (*in vivo*)	Mota et al. (2011)
Bisphenol A (BPA)	Deformation of seminiferous tubules, apoptosis in testes, and decreased spermatozoa in offspring	*In vivo* experiments in mice	Wei et al. (2019)
Diethylhexyl phthalate (DEHP)	Reduced quality parameters	Dog (*in vivo*)	Lea et al. (2016)
Polychlorinated biphenyl 153 (PCB 153)	Influenced sperm quality index	Dog (*in vivo*)	Lea et al. (2016)
1,2-Dibromo-3-chloropropane (DBCP)	ROS inducer, reduced germ cell viability, and eventually, sperm production	Human (*in vitro*)	Easley IV et al. (2015)
2-Bromopropane (2-BP)	Overwhelmed ROS, apoptosis of germ cells, and minimized sperm viability	Human (*in vitro*)	Easley IV et al. (2015)
Phthalate mixtures	Prenatal exposure altered the testicular steroidogenic gene	Mice (*in vivo*)	Barakat et al. (2019)

Keeping in view the aforementioned information, it is pivotal to review environmental toxicant-regulated reproductive toxicology. Emphasis is also placed on the mechanistic approaches to how redox sensors are involved in reproductive toxicity and their evidence in different animal models. While information about reproductive toxicants remains limited, there is a need to consolidate state-of-the-art knowledge to enhance our understanding and develop targeted therapeutic approaches in the near future.

### Disruption in the redox status

Reactive oxygen species (ROS) are the byproducts of cellular metabolism, produced from a variety of sources. The oxygen molecules are effectively used in mitochondria. Oxygen leakage is responsible for the production of superoxide anion radicals, which can react with other radicals to produce nitric oxide from reactive nitrogen species (RNS). RNS are nitrogen-comprising compounds such as peroxynitrite anion, nitroxyl ion, and nitric oxide. Irrespective of their physiological role, excessive formation of RNS causes nitrosative stress, which can have adverse effects on the male reproductive system (Sikka, 2001; Lee and Cheng, 2004; Pacher et al., 2007). Superoxide anion and hydrogen peroxide can form hydroxyl radicals. Similarly, the superoxide anion combines with nitric oxide (NO) to form peroxynitrite (Aon et al., 2012). Superoxide and byproducts of lipid peroxidation are radicals that are strong stimulators of mitochondrial uncoupling proteins, autophagic engulfment, and signaling molecules that have important functions in differentiation, adhesion, migration, and cell survival. The superoxide level was reported to be higher in mitochondria than in the cytoplasm (Cadenas and Davies, 2000). The superoxide anion is short-lived and membrane-impermeable, bearing a strong capacity to damage lipids, proteins, and mitochondrial DNA. However, superoxide breaks down into hydrogen peroxide either spontaneously or with the superoxide dismutase (SOD) enzyme. Hydrogen peroxide activates the redox-sensitive pathways in enormous cellular functions (Skakkebaek et al., 2001; Benjamin et al., 2017). Mitochondria encompass potent enzymatic and non-enzymatic antioxidant defenses. Glutathione is a strong intracellular thiol used in scavenging ROS, xenobiotics, and mitochondrial GSH, which constitutes 10%–15% of total cellular GSH (Lash, 2006). Once GSH is oxidized, it forms oxidized glutathione (GSSG), which can be reduced by glutathione reductase to maintain cellular redox status. The SOD enzyme dismutase superoxide into hydrogen peroxides and then converted into molecular oxygen and water through the catalase enzyme or glutathione peroxidase-1, seleno-enzyme. These enzymes convert hydrogen peroxide into water via the oxidation of GSH to GSSG (Cole-Ezea et al., 2012).

### Environmental toxicants and sperm quality

**FIGURE 1:**
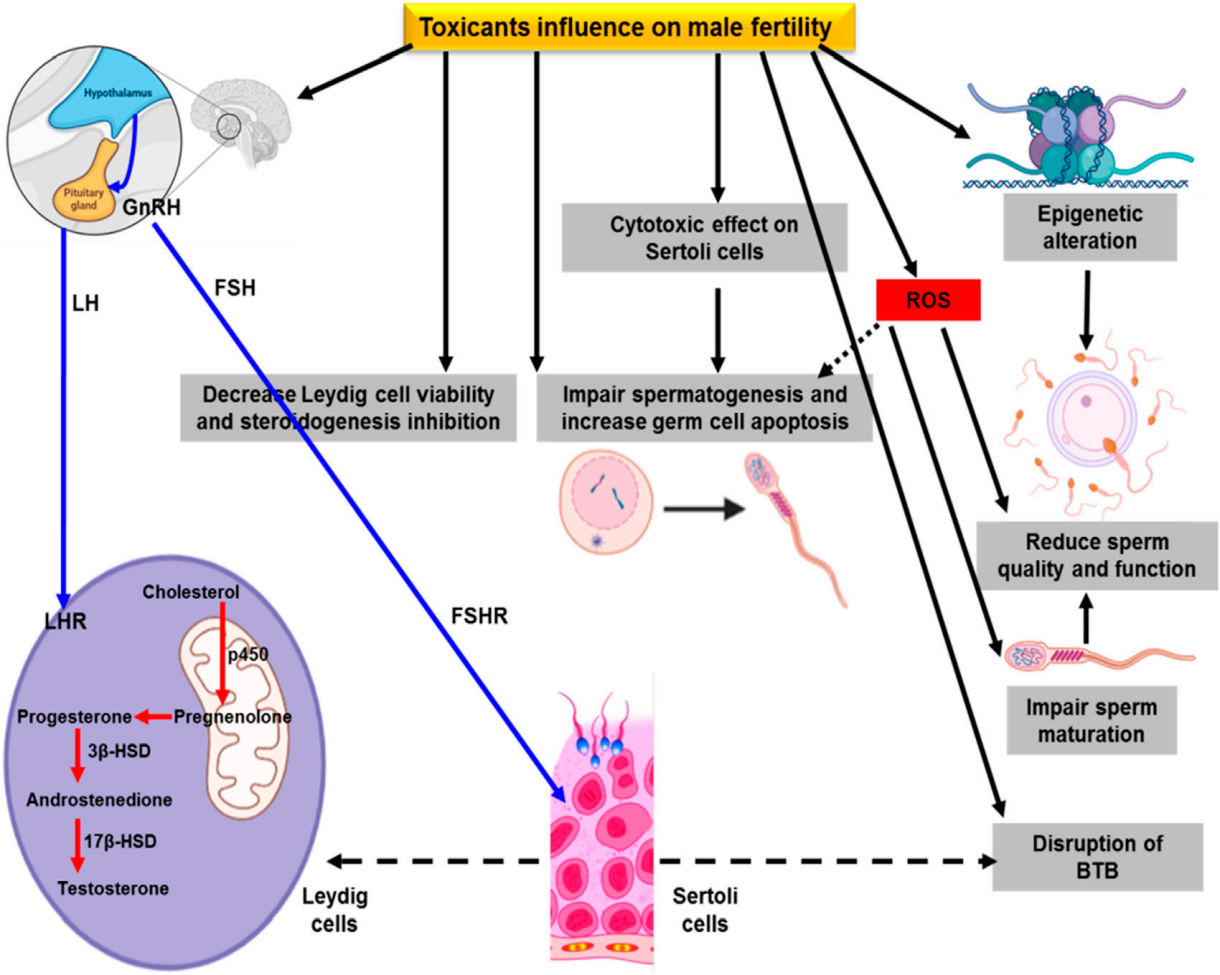
Effect of environmental toxicants on male fertility.

ROS are the byproducts of oxygen metabolism that are crucial for cellular homeostasis (Sies and Jones, 2020). Previous research has shown that environmental toxins cause oxidative stress via the overproduction of ROS. ROS plays a key role in the defense mechanism against a pathological milieu, but overwhelming production can have deleterious effects on tissues (Puppel et al., 2015). The function of ROS in male infertility is observed due to defective sperm production, whereas a greater amount of ROS damages the sperm plasma membrane and eventually causes sperm dysfunction (Kefer et al., 2009). The semen is well-equipped with enzymatic and non-enzymatic antioxidants, which coordinately work to ensure maximum protection from ROS effects. The main sources of ROS in the semen are mitochondria, immature sperm cells, leukocytes, and bacterial byproducts such as cytokines and bacterial and viral infections. It has been noted that ROS plays an essential role in sperm capacitation, acrosome reaction, mitochondrial stability, and sperm motility. Spermatozoa consist of poly-unsaturated fatty acids, which give them delicate appearances and make them extremely vulnerable to oxidative attack. Once the ROS damage to the sperm plasma membrane occurs, it is oxidized by lipid peroxidation; however, the cytoplasm contains low levels of enzymes that are unable to neutralize high levels of ROS (Walczak-Jedrzejowska et al., 2013). Sperm lipid peroxidation results in the loss of membrane integrity and enhances permeability, inactivation of cellular enzymes, DNA damage, and cellular apoptosis. As a consequence, it reduces sperm count, activity, and motility and causes abnormal morphology (Fraczek and Kurpisz, 2005). The effect of environmental toxicants on male reproduction is shown in Figure 1.

Male infertility can be assessed through the sperm quality index. Toxin exposure reduces sperm quality and enhances cryptorchidism in infants. In dogs, long-term reduced sperm quality was noticed due to the high risk of cryptorchidism. Lea et al. (2016) has reported the evidence of di (2-ethylhexyl), phthalate (DEHP) and organochloride in dog testes and in commercial feed. Polychlorinated biphenyl 153 (PCB153) and DEHP at various levels in humans and dogs resulted in DNA damage and reduced sperm motility (Sumner et al., 2019). Epidemiological evidence for PCB, triclosan, and bisphenol A (BPA) has reported adverse effects on seminal plasma, resulting in poor semen quality (Albert et al., 2018; Mantzouki et al., 2019). Wang et al. (2019) documented enhanced urinary phthalates that are linked to humans with low sperm quality due to a variation in metabolic seminal plasma.

Exposure of rodent spermatozoa to BPA resulted in reduced sperm quality parameters (Rahman et al., 2015). A higher level of tyrosine phosphorylation in sperm regulates protein-dependent kinase (PKA), which facilitates the acrosome reaction. Exposure to BPA alters fertility-related proteins that trigger immature acrosome reactions, leading to a decline in fertility and embryo survival (Rahman et al., 2015). In addition, BPA exposure resulted in undesirable effects on sperm features due to oxidative stress and DNA damage (Ikhlas and Ahmad, 2020). Other studies have reported that human spermatozoa, when exposed to dibutyl phthalate (DBP) and monobutyl phthalate (MBP), suppressed sperm tyrosine phosphorylation that takes part in sperm activeness (Xie et al., 2019). Exposure to DBP in treating inflammatory bowel disease (IBD) resulted in different mRNA expressions in sperm with oxidant production and DNA damage (Estill et al., 2019). Another compound, chlorothalonil, causes reduced boar sperm motility and enhanced apoptosis (Zhang et al., 2019a).

### Alteration in steroidogenesis by environmental chemicals

Previous literature has revealed that a variety of environmental toxicants alter reproduction via modifications in the hormonal system (McLachlan, 2016; Louis et al., 2018). In particular, toxicants influence estrogenic and anti-androgenic activities and eventually impair reproduction. It suggests that the physiology of hormones is influenced by environmental toxicants (Adoamnei et al., 2018; Rehman et al., 2018). For example, the anti-androgenic or anti-estrogenic properties of BPA influence hormonal balance and reduce semen production in humans with increased urinary BPA levels (Lassen et al., 2014). Disruption in the hormonal system due to toxicants is responsible for declining protein and steroid hormone levels in humans (Adamkovicova et al., 2014; Ren et al., 2020).

**FIGURE 2:**
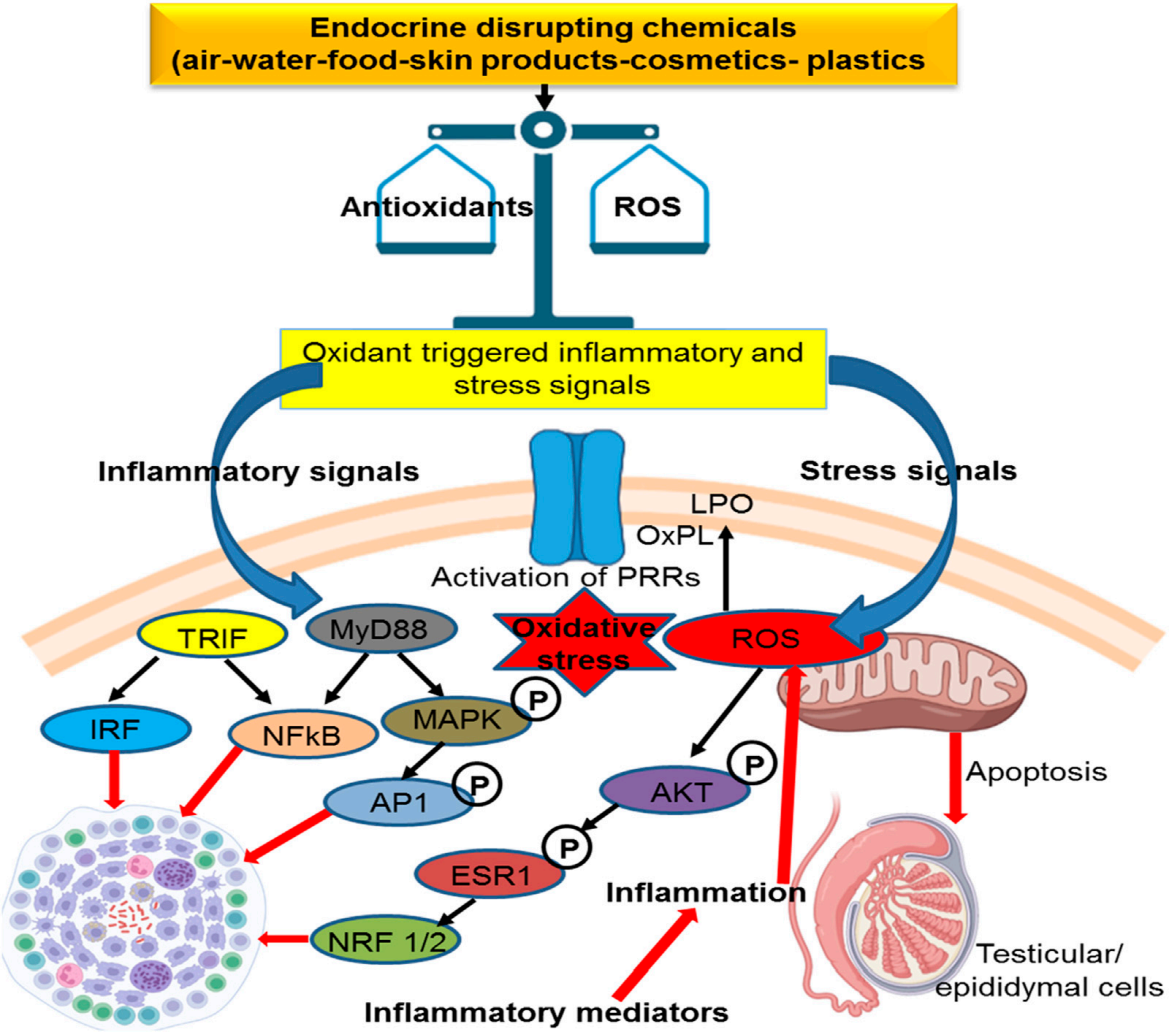
Mechanism of action of environmental toxicants on male fertility.

Environmental toxicants possess the ability to manipulate LH receptors (LHRs) and subsequently influence testicular steroidogenesis (Wang et al., 2017). For example, PFOS and PFOA bind with LH receptors and suppress testosterone synthesis (Foresta et al., 2018). Exposure to different environmental chemicals reduces LHRs, affects downstream signaling, and suppresses steroidogenesis enzymes (Pogrmic-Majkic et al., 2016; Wang et al., 2017). Reduced expression of StAR and suppression of the P450 SCC (mitochondrial cholesterol side-chain cleavage) enzyme is an efficient mechanism involved in the anti-steroidogenesis effect of environmental toxicants (Hong et al., 2016; Paul et al., 2017). Environmental toxicants directly suppress a variety of steroidogenesis enzymes but indirectly suppress them via oxidants (Saradha et al., 2008; Sheweita et al., 2016). Previous studies elaborate that oxidative stress is an indirect factor that negatively regulates male fertility due to environmental toxicants (Darbandi et al., 2018). An elevated concentration of ROS suppresses hormonal enzymes and triggers testicular apoptosis, affecting steroidogenesis and spermatogenesis (Quan et al., 2017; Shi et al., 2018). The molecular mechanism of toxins that influenced male reproduction is shown in Figure 2.

**FIGURE 3:**
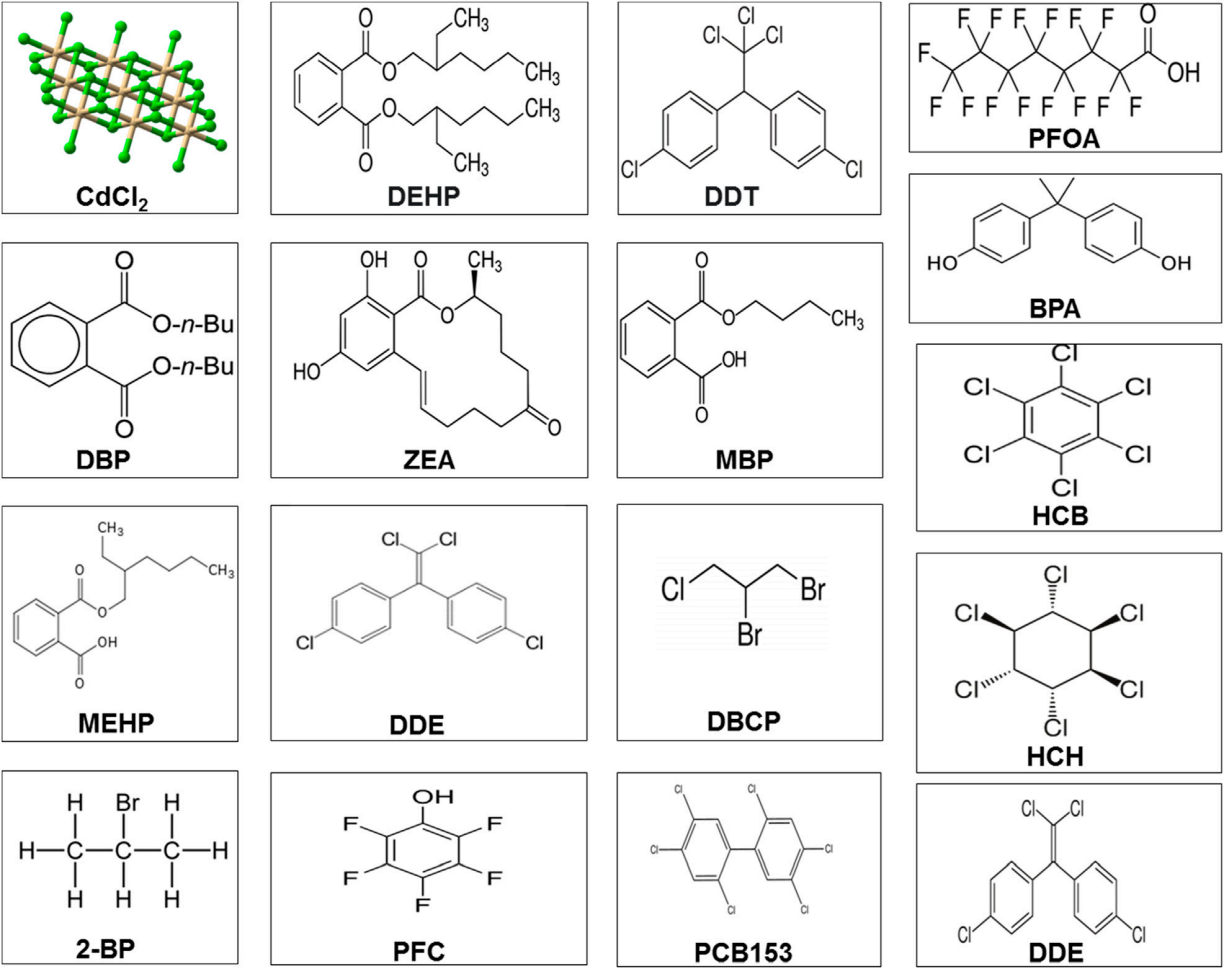
Chemical structure of different environmental toxicants. Abbreviations and categories Pesticides: dichlorodiphenyltrichloroethane (PFOA); 2-bromopropane (2-BP); perfluorochemical (PFC); hexachlorobenzene (HCB); dichlorodiphenyldichloroethylene (DDE); 1,2-dibromo-3-chloropropane (DBCP); perfluorochemicals (PFC); 2,2',4,4',5,5'-hexachlorobiphenyl (PCB 153); and dichlorodiphenyltrichloroethane (DDT) and its metabolite, dichlorodiphenyldichloroethylene (DDE). Phthalates or plasticizers: di(2-ethylhexyl) phthalate (DEHP); dibutyl phthalate (DBP); monobutyl phthalate (MBP); mono(2-ethylhexyl) phthalate (MEHP); monobutyl phthalate (MBP); and bisphenol A (BPA). Heavy metals: cadmium chloride (CdCl2). Mycotoxins: zearalenone (ZEA).

Leydig cells contribute to testosterone synthesis via communication with LH (Odermatt et al., 2016). Testosterone is the dominant male hormone accountable for sexual vigor and other secondary sexual characteristics. Disruption in Leydig cell viability affects testicular steroidogenesis and may result in disturbances in the endocrine functions of spermatogenesis and, thus, impair fertility. Mouse Leydig cells exposed to Aroclor 1242 resulted in decreased viability of Leydig cells and influenced testosterone synthesis by suppressing HSD and 17β-HSD enzymes (Aydin and Erkan, 2017). Organochlorines are known to modify the testicular StAR protein androgen-binding protein and stimulate 3β-HSD and 17β-HSD with increased H_2_O_2_ in adult male rats (Saradha et al., 2008). It has been shown that these substances suppress steroidogenesis, which affects Sertoli cell function and produces oxidant products (Saradha et al., 2008). Long-term exposure to arsenite causes immune-stimulant responses in the testis, which influences steroidogenic metabolism (de Araújo Ramos et al., 2017). The chemical structures of the environmental toxicants are displayed in Figure 3.

### Environmental chemicals and male reproductive organs

The environmental toxicants affect various reproductive organs, but the testis is considered the most vulnerable target. Spermatogenesis and steroidogenesis are hampered by the increased expression of estrogen receptors in the testis, which may affect the testis’ ability to withstand environmental toxins (Krzastek et al., 2020). Fenvalerate disrupts the spermatogenic cycle in rats close to puberty through germ cell apoptosis (Zhang et al., 2018). Reduced germ cell viability has been confirmed by *in vitro* exposure to DBCP and 2‒BP in human spermatogenic models through oxidant products and apoptosis (Easley IV et al., 2015). Perfluorinated compounds (PFCs) were examined in a study by [68], and a connection between blood and semen damage was found. Research has demonstrated alterations in sperm indices in conjunction with further sperm damage in PFC-positive individuals. It has, therefore, been discovered that PFC can have detrimental effects on DNA and disrupt meiotic segregation by influencing spermatogenesis (Governini et al., 2015).

The Sertoli cells are localized in the testes, nurturing germ cells across the sperm cycle. A particular ratio of Sertoli and germ cells is required for spermatogenic sustainability and metabolism (Rebourcet et al., 2017). Exposure to cypermethrin resulted in an alteration in the epithelial size and dedifferentiation of testis Sertoli cells and, thus, negatively impacted Sertoli cell functions in mice (Rodríguez et al., 2017). In addition, pollutants impact the molecular communication between germ cells and Sertoli cells (Gao et al., 2015). The degradation of Sertoli–germ cells in rats subjected to endosulfan and organochlorine led to oxidative stress and deteriorated gamete quality (Rastogi et al., 2014). Earlier studies revealed that DDE reduces testicular mitochondrial bioenergetic indices and their role in male fertility (Rastogi et al., 2014).

Rats exposed to BTB and cultivated Sertoli cells have been shown to be affected by exposure to Aroclor 1254 (a commercial PCB combination), which influences junctional proteins via the MAPK pathway. Using *in vitro* and *in vivo* models of PFOS on BTB, increased BTB permeability, ATF2 phosphorylation, and matrix metalloproteinase 9 expression were observed upon PFOS induction, together with decreased levels of occludin and connexin 43. These findings show that p38/ATF2/MMP9 works against PFOS-mediated BTB disruption (Qiu et al., 2016). Human Sertoli cells treated with BPA and cadmium chloride (CdCl_2_) were observed in influencing Sertoli cell adhesive function through changes in the F-actin network (Xiao et al., 2014). Another study on Sertoli cells in humans exposed to MBP showed decreased expression of the androgen receptor (AR), occludin, ZO-1, and β-catenin (de Freitas et al., 2016). Thus, it shows that MBP alters BTB activity via the AR-dependent pathway (de Freitas et al., 2016). The results unveiled that junctional proteins are more vulnerable to the harmful effects of various environmental toxins. Dankers et al. (2013) demonstrated that BPA, TBBPA, DEHP, MEHP, PFOA, and PFOS influence ATP-associated transporters in BTB, indicating reduced levels of testosterone in mouse Leydig cells. The environmental toxicant-induced apoptotic mechanism potentiated by oxidative stress is shown in Table 2.

**TABLE 2:** Oxidative stress-involved mechanism of apoptosis induced by environmental toxins.

Toxin	Cell line/tissue	Genes and proteins	Possible mechanisms involved	Reference
Bisphenol A	Germ cells/Leydig cells	↑Fas, ↑FasL, and ↑caspase-3	The Fas signal pathway induces Leydig cells and germ cell apoptosis	Li et al. (2009a), Takahashi and Oishi (2003)
Di(2-ethylhexyl) phthalate	Germ cells	↑Cyt c and ↑MEHP	DEHP metabolism disrupts OS in mitochondria	Kasahara et al. (2002)
Mono(2-ethylhexyl) phthalate	Germ cells	↑NF-kB and ↑sTNFα	Promotes Fas signals and upregulates Sertoli cell FasL expression	Yao et al. (2009)
Cadmium	Primary pig Sertoli cells	↓ Cell proliferation, ↑ DNA damage, and ↑ apoptosis	MAPK-activated p38	Wong and Cheng (2011)
Cadmium	Rat testis	↑ Association between FAK, occludin, and ZO-1	MAPK p38 and JNK	Wong and Cheng (2011)
Cadmium	Rat Sertoli cell–gonocyte coculture	↑ Apoptosis and ↑ detachment of gonocytes from Sertoli cells	JNK and p38	Wong and Cheng (2011)
Bisphenol A	Rat testis and primary rat Sertoli cells	↓ Occludin and N-cadherin on the Sertoli cell surface, ↓ TJ, AJ, and gap junction protein levels, and delay in gap junction communication	ERK	Wong and Cheng (2011)
Bisphenol A	Rat testis	↑ Raf1 and phosphotyrosine proteins and ↑ in spermatogonia and Leydig cell number	ERK	Wong and Cheng (2011)
Bisphenol A	Rat Leydig cells (R2C cells)	↑ aromatase, cyclooxygenase-2, prostaglandin E2, ↓ testosterone, and activation of protein kinase A and B	ERK, JNK, and p38	Wong and Cheng (2011)

### Mitochondria and environmental toxicants

The mitochondria are an essential cellular component involved in energy metabolism, regulating signaling pathways, the generation of metabolites, calcium storage, steroid synthesis, and apoptosis (Coffman et al., 2009; Marchi et al., 2018). Therefore, they are an essential entity for cell survival and homeostasis.

The respiratory chains in mitochondria are composed of five transmembrane enzyme complexes; they work together with electron transfer carriers, ubiquinone, and cytochrome c to generate ATP during oxidative phosphorylation (Brand and Nicholls, 2011). In this process, electrons leak from complexes I, II, and III and react with oxygen to generate superoxide. The superoxide radical is converted into hydrogen peroxide through the superoxide dismutase enzyme. Hydrogen peroxide and superoxide radicals are known as mitochondrial ROS (Dunn et al., 2015; Zia et al., 2022).

These complexes can easily be targeted by environmental toxicants that alter their expression levels and activities (Brand and Nicholls, 2011). During oxidative phosphorylation, the complexes help maintain the electrochemical gradient through a sequence of redox reactions. The electrochemical gradient forms the mitochondrial membrane potential, which is compulsory for energy production. An interruption in these complexes perturbs electron transfer carriers or proteins and causes damage to membranes and external chemicals that can alter the membrane potential, which eventually influences ATP and provokes cell death (Sakamuru et al., 2016; Zorova et al., 2018).

Alteration in oxidative phosphorylation in mitochondrial defects, often known as downstream effects, was measured as a biomarker. The mitochondrial oxidation and generation of 8-OHdG are the key free radical inducers of DNA lesions (Ziech et al., 2010). Elevated levels of 8-OHdG are an indicator of DNA damage and are referred to as markers of mitochondrial dysfunction (Valavanidis et al., 2009).

Accelerated ROS production can threaten mitochondrial biomolecules, trigger mitochondrial DNA (mtDNA) mutations, change membrane permeability and structure, and alter calcium ion homeostasis (Gao et al., 2003; Blajszczak and Bonini, 2017). Once the mtDNA is influenced, it possesses less repair capacity than the nucleus (Phillips et al., 2014). When the persistent mtDNA damage is significant, it may have a downstream detrimental effect on the mitochondria.

The calcium concentration plays an essential role in regulating membrane potential, ROS homeostasis, and oxidative phosphorylation in mitochondria (Giorgi et al., 2018). As a consequence, disruption in the transfer of mitochondrial calcium changes ATP levels and downregulates mitochondrial metabolism, whereas increased levels of mitochondrial calcium indicate impairment of the electrochemical gradient (Xu et al., 2016; Giorgi et al., 2018). Oxidative stress is associated with toxicant-mediated calcium levels; a breakdown in the membrane potential leads to cell death (Xu et al., 2016). *In vitro* studies of calcium levels are used to estimate mitochondrial dysfunction, but it is not clear whether the mitochondrial damage occurs due to the toxicants (Brand and Nicholls, 2011).

### Redox dysregulation and teratogenesis

Various environmental and therapeutic chemicals are included in developmental toxicants. They vary in their structure, function, and usage and have diverse modes of action. Fascinatingly, most of these and other chemicals can serve as oxidants, producing either directly or indirectly ROS and other reactive compounds (Hansen, 2006).

However, the consequences of oxidative insults triggered by these entities have not been profoundly reported in developmental models. This concept of oxidative stress impairs redox pathways and other targets of oxidation. In developmental toxicology, the majority of the literature has documented harmful consequences and toxicities that come from the overproduction of oxidants or depletion of the antioxidant system due to oxidative stress. Mounting evidence from seminal studies has demonstrated that ROS is involved in developmental toxicant production such as phenytoin, hydroxyurea, and ethanol (Liu and Wells, 1995; Miller-Pinsler et al., 2015). The application of 8-OHdG comprises the impact on DNA repair mechanisms (Shapiro et al., 2016), cell cycle regulation (Petrova et al., 2018), and suppressor tumor gene function (p53), causing impairment of a developmental program (El Husseini et al., 2016). Mounting evidence has shown that birth defects are caused by oxidative stress during developmental program. This type of oxidative damage is mainly toxico–pathological, but redox homeostasis also plays an essential role in normal physiology (Pizzino et al., 2017).

Earlier, thalidomide was used for the treatment of serious diseases, and it could be used until safe therapeutic options were available (Kim and Scialli, 2011). However, its teratogenic effect has not been proven in rodents but only in rabbits. To find out the variance in species vulnerability, comparative studies of mouse and rabbit embryos exposed to thalidomide showed that rabbits had high oxidative stress markers (Parman et al., 1999). Treatment-exposed free radical traps and decreased deformities of the limb buds demonstrated that oxidative stress resulting from thalidomide is a primary mechanism of teratogenesis. Other evidence found that thalidomide-induced oxidative stress and its anti-angiogenic activity may cause teratogenicity (4, 5). Thalidomide is a sedative and has a teratogenic effect. It was identified that cereblon (CRBN), as a thalidomide-binding protein, is the primary cause of teratogenicity. The teratogenic effect starts when it binds to CRBN and suppresses related ubiquitin ligase activity (Ito et al., 2010).

Several environmental pollutants, comprising polycyclic aromatic hydrocarbons and pesticides, have been documented as metabolic disrupting chemicals (Ferm, 1977) and may result in mitochondrial dysfunction. Available evidence has shown an alteration in MRC-exposed toxins. A number of herbicides, fungicides, insecticides, and acaricides alter the function of MRC, leading to the formation of ROS and reduced ATP levels (Fischer and Bavister, 1993). For instance, pyrethroids reduce ΔΨm and diminish the expression of cytochrome c, hence decreasing the function of cytochrome c oxidase in rat brains (Fisher and Burggren, 2007). These can trigger the reduced complex-I activity of MRC linked with nigral dopaminergic neurodegeneration and microglial activation, as observed in Parkinson’s disease (Folmes and Terzic, 2015). Moreover, evidence of environmental toxins, mitochondrial function, and male reproduction needs further elucidation in different animal models.

### Environmental toxicants and redox signaling pathways

#### Fas/FasL signaling pathway

Fas/FasL is a signaling molecule, pivotal for the regulation of apoptosis. It is expressed in peripheral T and B lymphocytes, NK cells, mononuclear cells, fibroblasts, endothelial cells, epithelial cells, etc. However, the expression of FasL is restricted to activated T cells, NK cells, and testicular Sertoli cells (Chai et al., 2008). FasL is considered a marker of functional Sertoli cells (Suda et al., 1993; Ma et al., 2016), but other authors believed that FasL expression was limited to sperm cells (D'Alessio et al., 2001; Riccioli et al., 2003). Moreover, the Fas/FasL pathway is triggered by glucocorticoids in Leydig cell apoptosis (Gao et al., 2003). It is believed that environmental toxicants induce testicular pathology through the activation of Fas/FasL pathway. Moreover, such signals are engaged to influence the sensitivity of germ cells, steroidogenic function, and cytokine metabolism regulated by Sertoli cells, and provoke the stimulation of the nuclear factor of activated T cells (NFAT) in Leydig cell apoptosis.

The apoptotic process is maintained by an optimal ratio of germ cells to Sertoli cells; thereby, fruitful spermatogenesis and fertility processes occur. At the adult stage, apoptosis is characterized in spermatocytes, depending on the balance between Bcl xL and Ba (Rodriguez et al., 1997). Testicular apoptosis is regulated via closely linked pathways in Sertoli cells, germ cells, Leydig cells, and numerous other signals. Such a phenomenon erases selective germ cells, which are damaged through different physiological and environmental triggers. In addition, apoptosis excludes senescent moribund spermatozoa through a phagocytic process (Aitken and Baker, 2013). Unfortunately, environmental toxicants with a specifically low level of heavy metal exposure exert negative effects on male reproductive function (Wirth and Mijal, 2010). Recent findings have revealed that an imbalance between cell survival and apoptosis due to disease or environmental factors adversely influences spermatogenesis, resulting in oligospermia, azoospermia, and hematospermia (Almeida et al., 2013). In addition, enhanced expression of Fas/FasL triggers apoptosis of germ cells in seminiferous tubule stages VII–VIII and IX–XII, when testes are exposed to environmental toxins (Zhao et al., 2011).

Microcystin activates the Fas/FasL signaling molecule via suppressing protein phosphatases 1 and 2A (PP1/PP2A), disturbing cell phosphorylation, and diminishing the cytoskeleton. This results in the stimulation and differential expression of transcription factors and proteins that contribute to cell differentiation, proliferation, and tumorigenesis, leading to abnormal cell proliferation, apoptosis, and necrosis (Chen et al., 2016). The vulnerability of germ cells is associated with the Fas/FasL signaling pathway, which can control germ cell apoptosis. Exposure to environmental toxins during embryonic development increases the adult stage anomalies in spermatogenesis, with minimal exposure to toxins during adulthood leading to increased death of germ cells through the activation of Fas/FasL signals (Traore et al., 2016). Toxic elements (MEHP and BLCO) activate matrix metalloproteinase-2 (MMP-2) by downregulating Sertoli cell tissue inhibitors of metalloproteinases-2 (TIMP-2), thereby degrading tumor necrosis factor-alpha (TNF-α) (Yao et al., 2009; Ebokaiwe et al., 2015). TNF-α communicates with the Sertoli cell to trigger the NF-kB response (Yao et al., 2007), thereby stimulating the expression of FasL and commencing germ cell apoptosis. Thus, at some level, the vulnerability of germ cells can be controlled via Sertoli cells (Yao et al., 2009).

#### NF-κB signaling pathways

NF-κB is a transcription factor that has been associated with apoptosis. It is normally localized in the cytoplasm (inactive form) and is surrounded by IκB proteins. Stressors stimulate NF-κB via the degradation of IκB proteins, allowing its translocation into the nucleus (Wang and Gao, 2006). NF-κB can have pro-apoptotic and anti-apoptotic activities in similar cell types; thus, its function is determined by the environment. NF-κB upregulates several genes, comprising Fas and death receptors 4, 5, and 6. Molina et al. (2005) documented that rats exposed to lindane caused NF-κB stimulation within 24 h in testicular germ cells, while maximum activity of Fas expression was not observed until 72 h post-exposure. Such activity reveals that Fas expression is enhanced due to NF-κB upregulation, suggesting a pro-apoptotic function of NF-κB. Previous findings have revealed that NF-κB may act as a suppressing agent in glucocorticoid-mediated apoptosis. In a study of rat Leydig cells, where NF-κB suppressed CORT-triggered apoptosis, NF-κB overexpressed cells were less prone to CORT-induced apoptosis; cells treated with PDTC (NF-κB inhibitor) exerted an increased level of CORT-induced apoptosis (Wang and Gao, 2006). Hence, NF-kB serves as an anti-apoptotic agent in receptor-triggered apoptosis. The exposure to MEHP induced a testicular NF-κB response, indicating the NF-κB significance in germ cell apoptosis. Exposure to MEHP induced various localization patterns in the rat testis. However, increased stimulation of spermatocytes was noticeable due to germ cells, which are at the meiotic stage and most vulnerable to MEHP damage (Rasoulpour and Boekelheide, 2005).

Mitogen-activated protein kinases (MAPKs) are monitoring proteins that act as signaling molecules triggered by external stimuli. Extracellular signal-regulated kinases (ERKs) are signaling molecules that take part in the functioning of spermatogenesis and Sertoli cells (Li et al., 2009c). The activation of ERK1/2 suppresses the function of Sertoli cells and enhances testicular apoptosis (Takahashi and Oishi, 2003; Fiorini et al., 2004). Protein kinase B (AKT) mediates oxidative stress via the regulation of cell growth, cell survival, cell proliferation, and inflammation, including immune reactions. MAPK and AKT are both directly involved in the phosphorylation of NF-ĸB and translocation into DNA, thereby causing the translation of relevant genes (Dalsenter et al., 1997). Spermatogenesis and Sertoli cell function are governed by NF-ĸB. Its stimulation resulted in spermatogenic defects both in humans and mice (Kuiper et al., 1998; Saradha et al., 2008). Exposure to arsenic causes the activation of ERK/AKT/NF-ĸB signals in different cells (Gaido et al., 1999; Anway et al., 2005). Exposure to sodium arsenite (1, 5, or 25 mg/L for 6 months) promotes the expression of ERK1/2, IKKγ, PI3K, AKT, and NF-ĸB, along with enhanced phosphorylation of ERK/AKT levels in the testes of rats. Thus, it leads to reproductive toxicity via the stimulation of ERK/AKT/NF-kB signaling (Peter, 2000).

#### MAPK signaling pathway

MAPK is a signaling pathway activated in response to different environmental toxicants. Three types of MAPKs, namely, ERK, JNK, and p38 are stimulated in the testis following exposure to environmental toxicants. MAPKs are known to contribute to various male reproductive functions such as cell cycle progression, steroidogenesis, sperm hyperactivation, and acrosome reaction (Li et al., 2009b; Almog and Naor, 2010). Therefore, environmental toxicants influence MAPK functions and cause pathological effects in males. They enhance DNA damage and apoptosis and distort cellular junctions and steroidogenesis (Kim et al., 2010; Zhang et al., 2010). The MAPKs are stimulated when environmental toxicants induce oxidative stress in cells and tissues. Suppressing oxidative stress via N-acetyl cysteine, a free radical scavenger, reverts cadmium-induced MAPK stimulation (Xu et al., 2003; Chen et al., 2008). This process is partially mediated by the suppression of Ser/Thr protein phosphatases 2A (PP2A) and 5 (PP5) through oxidative stress, which leads to enhanced phosphorylation of MAPK (Chen et al., 2008). Unfortunately, cadmium triggers the expression of MAPK phosphatase-1 (MKP-1) (Kim et al., 2008), the main suppressor of MAPK activation. It indicates the beneficial effects of MKP-1 via the suppression of protein phosphatases PP2A and PP5, leading to an increase in MAPK signaling, followed by exposure to environmental toxicants. Moreover, the stimulation of ERK can result in the phosphorylation of c-Src, FAK, and paxillin in the oxidative scenario, suggesting that MAPKs may be one of the upstream targets to stimulate non-receptor tyrosine kinases (Alderliesten et al., 2007).

#### PI3K/c-Src signaling pathway

Exposure to diverse environmental toxicants has been documented to induce testicular oxidative stress (Dhanabalan and Mathur, 2009; Liu et al., 2009). The development of OS enhances epithelial and endothelial permeability by impairing tight junctions and adherent junctions of cells (Sandoval and Witt, 2008; Lucas et al., 2009). Previous studies have documented that PI3K plays an essential role in regulating junction interruptions triggered by oxidative stress. Once it is challenged via oxidative stress, a regulatory subunit of PI3K p85 binds from the cytosol to the cell-to-cell interface (Qin and Chock, 2003; Sheth et al., 2003). Activation of PI3K subsequently stimulates a non-receptor tyrosine kinase, c-Src (Basuroy et al., 2010). In the testis, c-Src is mainly localized and specific at the blood–testis barrier and endoplasmic specialization (ES) (Box 1), related to connexin 43/plakofilin-2 and β1-integrin/lamininα3β3γ3 protein complexes specific to their cell junctions (Yan and Cheng, 2006; Lee et al., 2009a). Stimulation of the PI3K/c-Src signaling pathway due to oxidative stress from environmental toxicants may impair testicular function due to toxicants. Early research has shown that c-Src kinase activity in the testis indicates the harmful effects of 2,3,7, and 8-tetracholordibenzo-p-dioxin (El-Sabeawy et al., 1998). Moreover, increased levels of c-Src have also been reported in the testis, through cadmium exposure in rodents, showing that c-Src is stimulated against numerous environmental toxicants (Wong et al., 2004; Siu et al., 2009).

In epithelial cells, FAK is a substrate of c-Src, and FAK-Src is responsible for the regulation of various physiological and pathological cellular responses (Brunton and Frame, 2008; Bolós et al., 2010). FAK is a downstream regulator of the PI3K/c-Src pathway in oxidative stress-augmented junction interference (Sen et al., 2007; Basuroy et al., 2010). It is known that membrane translocation and stimulation of PI3K and c-Src through oxidative stress initiate FAK phosphorylation. Such a process enhances the tyrosine phosphorylation of junction proteins via FAK to change the adhesive function of protein complexes. Intriguingly, factors such as cell type, source of ROS, and duration of exposure can phosphorylate the FAK pathway (Weinberg et al., 2001; Alderliesten et al., 2007). At the beginning of oxidative stress, FAK is stimulated via c-Src to induce undesirable phosphorylation of junction proteins at the cell-to-cell interface. This causes the rearrangement of proteins in the cytosol and results in the distraction of TJ and AJ (Rao et al., 2002). Moreover, cell adhesion is further compromised by detachment of integral membrane proteins from their respective cytoplasmic adaptors (Rao et al., 2002; Sheth et al., 2003). Disassociation of the focal adhesion contract (Molina et al., 2005) and production of active aldehydes occurs during oxidative stress (Usatyuk et al., 2006), and thereafter, unstimulation of FAK occurs via dephosphorylation (Alderliesten et al., 2007). Such evidence indicates that FAK is a crucial regulator of TJ and AJ interruption during oxidative stress. Hence, the critical phosphorylation of FAK may exert a novel therapeutic target to guard the testis against oxidative damage.

#### Nrf2 signaling pathway

Nrf2 is a pivotal transcription factor that resides in living cells to protect against oxidative stress (Rubio et al., 2010). Nrf2 stimulation has been observed after exposure to sulforaphane (SFN), heavy metals (Shaw et al., 2019), pesticides (Li et al., 2011), and polycyclic aromatic hydrocarbons (Nguyen et al., 2010). Under physiological conditions, Nrf2 is localized in the cytoplasm via the Kelch-like ECH-associated protein 1 (Keap1). When Nrf2 is exposed to activators, it is isolated from Keap1, causing Nrf2 translocation from the cytoplasm to the nucleus and, ultimately, the antioxidant response element (ARE). Moreover, AREs are also responsible for phase II detoxification enzymes. Hence, stimulation of Nrf2/Keap1/ARE amplifies the expression of antioxidants and detoxifying enzymes such as SOD, catalase (CAT), GSH, NAD(P)H-quinone oxidoreductase 1 (NQO1), and heme oxygenase-1 (HO-1) (Chen and Chien, 2014).

Exposure to DEHP-triggered oxidative stress resulted in the upregulation of Nrf2 signaling (Amara et al., 2020). Zhang et al. (2019a) reported that DBP increased mitochondrial damage and germ cell death via the Nrf2-dependent pathway. Inhibition of the Nrf2/ARE pathway promoted DBP-triggered mitochondrial toxicity (Zhang et al., 2019b). They observed protein kinase endoplasmic reticulum kinase (PERK) through the manipulation of the Nrf2/ARE pathway. PERK causes phosphorylation of Nrf2 and, consequently, detachment of Nrf2/Keap1 to release genes. Suppressing PERK via its specific inhibitor causes inhibition of Nrf2, which increases DBP-prompted apoptosis and mitochondrial damage. Amara et al. (2019) demonstrated that DEHP caused cytotoxic effects in embryonic kidney cells (HEK-293) via inhibition of the Nrf-2/HO-1 pathway (Amara et al., 2019). Zhao et al. (2020) elaborated that DEHP exposure promoted Nrf2 activity via the generation of ROS in mouse testis, which aligns with the results of Tang et al. (2018). These authors showed that the activation of Nrf2 promotes Notch signal inhibition in the testis. Once Nrf2 was increased, Notch1 and hairy and enhancer of split 1 (Hes1) were decreased, causing interference in spermatogenesis and suppressing testosterone levels. Abd El-Fattah et al. (2016) documented that mRNA of Nrf2 and HO-1 was enhanced in DEHP compared to the control group in the rat testis (Abd El-Fattah et al., 2016). Suppressing Nrf2 signals was induced, followed by exposure to phthalates, which led to toxic damage in diverse cells and tissues comprising the Sertoli cells (Zhang et al., 2017). The activation of Nrf2 signals by DBP and DEHP in the reproductive system was not enough to decrease oxidative stress (Shen et al., 2015). Zhao et al. (2019) demonstrated that lycopene attenuated DEHP-induced Leydig cell damage, which may promote antioxidant capacity via regulation of the Nrf2 signaling pathway. Jiang et al. (2017) reported that SFN exerts a protective effect via the stimulation of Nrf2, along with its target genes, against DBP-induced sperm parameters and testicular cell apoptosis. SFN causes upregulation of Nrf2 and, thus, decreased DBP-augmented intracellular oxidative toxicity. According to Yang et al. (2018), SFN reversed cadmium-induced Sertoli cell toxicity in mice via stimulation of the Nrf2/ARE pathway, and, thus, oxidative damage and apoptosis were attenuated. In other studies, consumption of a plant-derived lutein compound mitigated arsenic-induced reproductive toxicity in a mouse model via upregulation of the Nrf2 pathway and, thus, prevented reproductive injury (Li et al., 2016).

### Epigenetic effects and environmental toxicants

According to earlier studies, epigenetic alteration may be a major factor in controlling how adversely environmental contaminants affect male fertility (Donkin and Barrès, 2018). The mechanism of epigenetics comprises methylation of DNA, alteration in histone proteins, and expression of miRNA genes (Dada et al., 2012; Muratori and De Geyter, 2019). In mice, exposure to zearalenone (ZEA) has been shown to disrupt the process of meiosis and signals that regulate spermatogenesis, resulting in lower semen features (Gao et al., 2015). In addition, mice exposed to ZEA had lower levels of methylation markers in DNA, like 5 mC and 5hmC, and higher levels of methylation in histone marker H3K27, as well as lower levels of testicular ER expression. Such evidence has documented the important connections between estrogen signaling and genetic and epigenetic pathways that regulate the negative effects of ZEA on spermatogenesis (Gao et al., 2015; Men et al., 2019). In F2 mice, exposure to nonylphenol triggered pathophysiological defects in the testis and epididymis that were known to be regulated by epigenetic programming upon exposure to nonylphenol (Kim et al., 2019). The biomarkers of male reproduction in response to toxicants are given in Table 3.

**TABLE 3:** Markers of male reproductive toxicity.

Marker	Expression	Reference
8-Hydroxy-2’-deoxyguanosine −8-OHdG	DNA damage due to DNA oxidation	Al-Hilli et al. (2018), Ommati and Heidari (2021)
Creatine	Excess creatine level in urine, indicating testicular damage	Zhang et al. (2014)
SP22 (sperm surface protein)	Relevance of toxins causes reduction in SP22 quality	El-Garawani et al. (2021)
Vitellogenins	Increased vitellogenin level shows toxic effects on male reproduction	Ge et al. (2016), Amthauer et al. (2021), Zhang et al. (2022)
Gene expression profiling (GEP)	Key marker of earlier risk of toxicity induction	Kier et al. (2004), Ommati and Heidari (2021)
D-aminolevulinic acid dehydratase (d-ALAD)	Biosynthetic enzyme and preliminary marker of lead poisoning that converts to porphobilinogen	Telišman et al. (2007)
Erythrocyte protoporphyrin (EP)	Exposure to heavy metals associated with age, alcohol, and smoking and lead serum biomarker	Telišman et al. (2007)
miR-27a	Inhibition of cysteine-rich secretory protein 2 (CRISP2) causes reproductive infertility	Zhou et al. (2017)
miR-34c-5p, miR-122, miR-146b 5p, miR-513a-5p, miR-374b, miR-509–5p, and miR181a	Reduced levels linked with azoospermia and asthenozoospermia and disruption of sperm regulation	Wang et al. (2011), Kong et al. (2012), Anyanwu and Orisakwe (2020)
piR-31704 and piR-39888	Key genotoxic factors and implied as an indicator of low sperm count	Cui et al. (2018), Anyanwu and Orisakwe (2020)

Animals exposed to environmental toxicants can pass epigenetic changes through generations (Rothstein et al., 2017). DBP exposure during embryonic development disrupts testicular activity in F1 and F3 generations by altering Sertoli cells and the spermatogenic process. Global DNA hypomethylation in the offspring was altered by DBP exposure (Yuan et al., 2017). Similarly, a short pregnancy exposure to atrazine triggered diverse DNA methylation in F1–F3 generation spermatozoa (McBirney et al., 2017). A large number of studies have reported that transgenerational inheritance of epigenetic modifications takes place once the embryo is exposed to environmental contaminants like chlordecone, DDT, vinclozolin, and DEHP, resulting in a negative impact on testes and semen index in F1–F3 offspring (Maamar et al., 2019; Skinner et al., 2019).

## Conclusion

Environmental chemicals such as pesticides, insecticides, heavy metals, and phthalates are toxic chemicals used worldwide for agricultural and other purposes. However, high levels of these toxins from human activities cause adverse effects on male fertility. The synthetic origin of phthalates is widely used in the plastic industry. They have a similar structure to steroid hormones. Available evidence shows that phthalates and other environmental toxicants interact with normal spermatogenesis and lead to testicular atrophy, oxidative stress, and DNA damage. It also interferes with steroidogenic pathways, resulting in decreased testosterone levels and Insl-3 production via fetal Leydig cells that induce cryptorchidism. It is well known that all these chemicals disturb mitochondrial metabolism, where CYP enzymes are involved. All these environmental chemicals influence male reproduction by disrupting the HPG system, the testes, the spermatogenic process, epididymal maturation, the antioxidant–antioxidant balance, and epigenetic regulation. In response to that, a reduction in the sperm quality index has been reported to cause male infertility. Considering previous studies, it is known that mitochondria and redox signaling such as Fas/FasL, NF-κB, MAPK, PI3K/c-Src, and Nrf2 are the main regulators of male reproductive toxicity, which is evident due to environmental toxicants and poses a serious threat to male reproduction. All of this suggests that oxidative stress is not the primary cause of toxicity; it is produced as a consequence of toxicity. Additional investigation is needed to determine whether the combined effects of pesticides at low doses, whether through environmental exposure or dietary intake during male developmental stages, may play a role in a cascade of cellular, molecular, and hormonal processes, leading to adverse effects on the male reproductive system.
